# A successful vaginal birth after cesarean in a patient with uterine didelphys

**DOI:** 10.1515/crpm-2023-0005

**Published:** 2023-09-04

**Authors:** Samantha Gobioff, Michael Plakogiannis, Amos Grünebaum

**Affiliations:** Department of Obstetrics and Gynecology, Lenox Hill Hospital – Northwell Health/Zucker School of Medicine, New York, NY, USA

**Keywords:** TOLAC, VBAC, Uterine Anomaly

## Abstract

**Objectives:**

With increasing rates of cesarean delivery across the United States, a trial of labor after cesarean (TOLAC) is a reasonable alternative for qualified candidates. Although Müllerian anomalies are associated with a variety of adverse pregnancy outcomes, there is little existing data regarding TOLAC in these patients. We present a case of a patient with a didelphys uterus who achieved a successful vaginal birth after cesarean section (VBAC) in the setting of labor augmentation.

**Case presentation:**

Our patient is a 32-year-old G4P1021 (Gravida 4 Para 1,021–1 term delivery, 0 preterm deliveries, 2 abortions, 1 living offspring) who presented at 8 weeks of gestation with a known history of a didelphys uterus. Her obstetrical history was significant for a prior low-transverse cesarean section at term. All four of her pregnancies were located in the right uterine horn. At 39 weeks 3 days of gestation she presented in early labor and requested a TOLAC. She received an epidural, a cervical ripening balloon was placed, and she was started on pitocin. She pushed to deliver a viable infant. The patient’s postpartum course was uncomplicated, and she was discharged home on postpartum day two.

**Conclusions:**

Müllerian anomalies are associated with several poor pregnancy outcomes including increased rates of PPROM, preterm delivery, FGR, and malpresentation necessitating a cesarean section. Our patient required augmentation of her labor but was ultimately able to achieve a successful VBAC with a healthy neonate. She represents an understudied population of patients with uterine anomalies who not only can have favorable pregnancy outcomes but may even be able to safely achieve a VBAC.

## Introduction

With increasing rates of cesarean delivery across the United States (approximately 32 % in 2021), a trial of labor after cesarean (TOLAC) is a reasonable alternative for qualified candidates [1]. On an individual patient level, vaginal birth after cesarean (VBAC) is associated with decreased maternal morbidity and mortality, while on a population level, it decreases rates of cesarean section. In fact, as a Level A ACOG recommendation, TOLAC should be offered to most women with one previous cesarean delivery with a low-transverse incision [2].

The widely used vaginal birth after cesarean calculator from the Eunice Kennedy Shriver National Institute of Child Health and Human Development (NICHD) is a tool many practitioners use to assess how likely a TOLAC candidate is to succeed and can be utilized in a shared decision-making conversation [3]. Criteria include maternal age, height, weight, prior obstetric history, history of arrest as the indication for prior cesarean, and treated chronic hypertension. Interestingly, Müllerian anomaly is not currently one of the criteria included and there is very little data regarding TOLAC in these patients. A 2007 retrospective study in AJOG showed that the rate of VBAC was significantly lower in patients with Müllerian anomalies than in patients with a normal uterus – 37.6 vs. 50.7 %, respectively [4].

The prevalence of uterine malformations is estimated to be 7 % of the general population and 18 % of those with recurrent pregnancy loss [5]. Because Müllerian anomalies are relatively uncommon, we were only able to find two prior reports of a VBAC associated with a uterine Müllerian anomaly – a 2015 case presentation of a patient with a didelphys uterus requiring vacuum assistance as well as a 2019 case presentation of a patient with a didelphys uterus who achieved a successful VBAC with a torn vertical uterine septum [6, 7]. We present a case presentation of a patient with a didelphys uterus without a vaginal septum who achieved a successful VBAC in the setting of labor augmentation with a cervical ripening balloon and pitocin. Informed consent was obtained from the individual included in this case presentation.

## Case presentation

**Figure 1:**
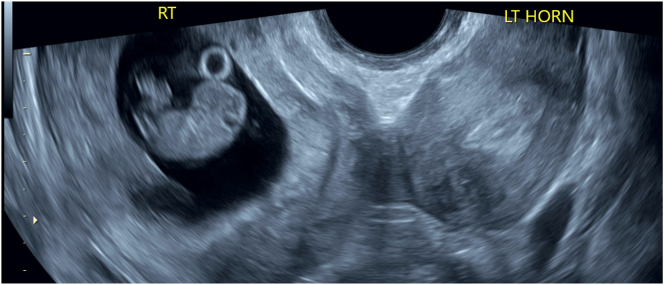
Dating ultrasound at 8 weeks gestational age showing a fetus in the right uterine horn and an empty left uterine horn.

Our patient is a 32-year-old G4P1021 (Gravida 4 Para 1,021–1 term delivery, 0 preterm deliveries, 2 abortions, 1 living offspring) with a body mass index of 37 who initially presented at 8 weeks of gestation for her dating ultrasound, which depicted a fetus in the right uterine horn and an empty left uterine horn (Figure 1). The patient had a known history of a didelphys uterus with two uteri and two cervices diagnosed several years prior on pelvic ultrasound. No additional imaging was obtained to evaluate for renal anomalies, which limited a complete antepartum evaluation, as renal tract malformations are highly associated with Müllarian anomalies. Her obstetrical history was significant for a prior low-transverse cesarean section at full term for arrest of dilation at 1.0 cm, a dilation and evacuation for a fetus with polycystic kidney disease and secondary anhydramnios, and a medical termination of pregnancy. All four of her pregnancies were located in the right uterine horn. Throughout her antepartum course, fetal growth was within normal limits with consistent cephalic presentation.

At 39 weeks 3 days of gestation she presented in early labor at 1.0 cm dilated, 80 % effaced, at −2 station. She denied incisional pain. Her VBAC score was calculated as 35 %. She requested a TOLAC, was counseled appropriately regarding the risks and benefits, and was admitted for labor augmentation. The fetal heart rate tracing on admission was Category I with approximately three contractions in ten minutes on tocometer. She received an epidural and vaginal exam was repeated. A rudimentary cervix was palpated on the patient’s left with a slightly dilated cervix identified on the patient’s right, through which the cervical ripening balloon was placed. Both the uterine and vaginal balloons were inflated with 80 cc of normal saline. Pitocin was also started at that time.

The balloon was in place for 11 h, at which time she was 4.0 cm dilated, 50 % effaced, at −2 station with the cervix still notably on the patient’s right. Her membranes were artificially ruptured at that time for clear fluid. She became fully dilated after 22 h on pitocin. She pushed for 90 min with a Category II tracing secondary to variable decelerations. Right mediolateral episiotomy was cut to expedite delivery of a viable female infant from LOA position with 1-and 5-min APGAR scores of 6 and 9, respectively. Birthweight was 3225 g (25.5th percentile). Retained placenta was manually extracted with a total estimated blood loss of 400 cc. The episiotomy was repaired with 2–0 vicryl in the standard fashion. The patient’s postpartum course was uncomplicated and she was discharged home on postpartum day 2.

## Discussion

Uterine didelphys is relatively rare, constituting approximately 7.8 % of Müllerian duct anomalies [4]. Müllerian anomalies are generally associated with poor pregnancy outcomes. In a retrospective cohort study in 2011, Hua et al. evaluated the relationship between congenital uterine anomalies and adverse pregnancy outcomes in 203 patients with Müllerian anomalies [8]. They found that the presence of a uterine anomaly was associated with a 7-fold increased risk of preterm birth less than 34 weeks of gestation and a 6-fold increased risk in preterm birth less than 37 weeks of gestation. The women with uterine didelphys had a higher proportion of preterm birth in general compared to any other subgroup. Uterine anomalies were also associated with a 3-fold increased risk of preterm premature rupture of membranes (PPROM). Of note, there were also statistically significant increased rates of placenta previa (OR 5.8), placental abruption (aOR 3.1), and fetal growth restriction (FGR) (aOR 2.0). Although didelphic uterus is uncommon, pregnancy outcomes are better when compared to septate or unicornuate uterus, with approximately 60–70 % resulting in a viable pregnancy [6].

One of the major indications for repeat cesarean section among patients with Müllerian anomalies is malpresentation, with an approximate 60 % malpresentation rate compared to 14 % in patients with a normal uterus [4]. For those with fetuses in vertex presentation, one of the theoretical concerns regarding TOLAC, especially in patients with Müllerian anomalies, is risk of uterine rupture. Erez et al. studied rates of uterine rupture as a primary outcome in patients attempting a VBAC in those with Müllerian anomalies, 8 % of whom had diagnosed uterine didelphys, and those with a normal uterus and did not find an increased risk of uterine rupture associated with Müllerian anomalies [4]. Importantly, compared to controls, patients with Müllerian anomalies who achieved a VBAC had no differences in the incidence of perinatal mortality, low 1- and 5-min APGAR scores, or peripartum complications.

Müllerian anomalies are associated with several poor pregnancy outcomes including increased rates of PPROM, preterm delivery, FGR, and malpresentation necessitating a cesarean section [8, 9]. Our patient had a low starting VBAC score of 35 %, independent of her didelphys uterus. However, after thorough counseling she decided to proceed with a trial of labor after cesarean section. She required augmentation of her labor, including a cervical ripening balloon and pitocin but was ultimately able to achieve a successful VBAC with delivery of a healthy neonate. She represents an understudied population of patients with uterine anomalies who not only can have favorable pregnancy outcomes but may even be able to safely achieve a VBAC.
